# Loneliness and depression among men in Poland: cross-sectional study

**DOI:** 10.3389/fpubh.2025.1539822

**Published:** 2025-06-17

**Authors:** Beata Dziedzic, Ewa Kobos, Katarzyna Przylepa, Anna Idzik

**Affiliations:** ^1^Department of Development of Nursing, Social and Medical Sciences, Faculty of Health Sciences, Medical University of Warsaw, Warsaw, Poland; ^2^Department of Basic Nursing and Medical Teaching, Chair of Development in Nursing, Faculty of Health Sciences, Medical University of Lublin, Lublin, Poland

**Keywords:** anxiety, depression, irritability, loneliness, men

## Abstract

**Introduction:**

Mental well-being is defined as subjective feeling characterized by an emotional and cognitive evaluation of one’s life that may could lead to high life satisfaction and low levels of negative emotions. Research findings confirm that individuals with an elevated level of loneliness often face mental health issues. Loneliness is recognize as an important potential predictor of depressive symptoms, anxiety, and suicidal thoughts. As mental health concerns are a serious crisis in many countries around the world, it is important to conduct research aimed at identifying those affected by this problem. Due to the reluctance of some men to seek professional health care, there is a need for screening tests to assess the risk of anxiety, depression and level of loneliness in this gender. The aim of the study was to assess the prevalence of anxiety symptoms, depression, irritability and assess the level of loneliness among men.

**Methods:**

The study was conducted on a representative sample of 438 men who completed a survey through an online portal using the Computer-Assisted Web Interviewing (CAWI) technique. The Hospital Anxiety and Depression Scale (HADS-M) and the UCLA Loneliness Scale (R-UCLA) were used to assess mental well-being. These scales assess only some aspects of mental well-being and are used in screening tests. The average age of the participating men was 45.61 ± 15.64 years.

**Results:**

On the HADS-M scale, participants scored an average of 13.91 ± 9.35 points. Anxiety clinically relevant symptoms were identified in 21.91% of the participants on the anxiety subscale, and depressive clinically relevant symptoms in 12.55% on the depression subscale. On the loneliness scale, participants scored an average of 40.50 ± 10.78 points, indicating moderate level of loneliness. A moderately high and very elevated level of loneliness was found in 21.00 and 2.30% of the participants, respectively.

**Conclusion:**

In this study, one fifth of the participants experienced anxiety clinically relevant symptoms, and every tenth man demonstrated depressive clinically relevant symptoms. Every fifth man experienced a moderately elevated level of loneliness. The main potential predictor of depression symptoms and higher levels of loneliness was the poor financial situation of the participants and a lack of financial decisiveness.

## 1 Introduction

Depression, classified within mental disorders, is a significant may contribute to of disability globally, impacting daily life, including relationships with family, friends and community and overall quality of life. Symptoms like sadness, fatigue, lack of interest in previously enjoyable activities, sleep and appetite disorders, increased exhaustion, and concentration issues can could lead to withdrawal from social, family, and work environments (1). Due to socially unacceptable behaviors in men such as direct admission of sadness and emotional weakness or sensitivity, depression symptoms in men might present differently, such as through anger, aggression, panic attacks, and might be coped with using psychoactive substances, gambling, or workaholism (2, 3). Untreated depression can may result in premature death due to physical health conditions and an increased suicide rate (4).

According to the World Health Organization (WHO), 5% of the global adult population suffers from depression (1). While women are more frequently affected (6%) than men (4%) (5), men are 3–4 times more likely to die by suicide and although no direct link has been found between depression and suicide, depression is one of the most important risk factors for suicide (6), despite attempting suicide less often than women. Men typically use more effective methods when it comes to taking their own lives. The mortality rate among 4,106 suicidal attempts among men was 13.91%, while among women, 4.836 suicidal attempts resulted in death in 4.05% (7). The higher suicide rate among men may be due to their choice of more lethal methods and a mentality valuing independence and reluctance to seek help, seen as a sign of weakness (8, 9). This contributes to many men with depression symptoms remaining undiagnosed (6).

In Poland in 2023, depression affected 2.8% of the adult population, including 2.3% of men, which is lower than the average in European Union countries (over 4% for the general population, including 3.1% for men) (10). However authors indicate a significant increase in the level of depression among working Poles in the period 2019–2022 (11). The “Health at Glance: Europe 2024” report suggests that differences between countries could be due to access to healthcare, awareness levels, and social acceptance, significantly impacting the ability to seek help and the number of diagnosed depression cases (12). It is estimated that over 75% of people in low- and middle-income countries do not receive treatment due to low investment in psychiatric care (5), lack of qualified staff, and social stigma, leading to shame and reduced help-seeking opportunities. Therefore, implementing preventive measures beyond the healthcare sector is crucial in identifying individuals at risk of mental health issues. WHO experts call for expanding care for people with anxiety and depression by enhancing identification efforts (13). The WHO report on the comprehensive mental health action plan for 2013–2030 emphasizes the need for attitude changes and actions to promote and protect mental health and provide care to those in need (14).

Mental health issues are complex, with anxiety disorders often co-occurring in individuals suffering from depression. It is estimated that about 85% of patients with depression symptoms also experience anxiety (15), and 90% of those with anxiety disorders suffer from depression (16). Additionally, high levels of loneliness are frequently associated with mental health concerns. Loneliness can predict the likelihood of depression, anxiety, suicidal thoughts, and dementia (17–23). Loneliness has been identified as one of the key predictors of depression, anxiety, and suicidal ideation. An increasing number of studies suggest that the relationship between loneliness and depression is reciprocal and dynamic. A 12-year longitudinal study of individuals aged 50 and over confirmed that higher levels of loneliness at the beginning of the study were associated with greater severity of depressive symptoms in subsequent years (24). Loneliness can could lead to depression, but depression may also deepen social isolation and the sense of loneliness (25, 26). The vicious cycle of loneliness model describes mechanisms through which loneliness intensifies cognitive biases, leading to withdrawal from social relationships, which in turn increases the risk of depression. On the other hand, depressive symptoms (e.g., loss of energy, anhedonia, negative self-perceptions) can reduce motivation to maintain interpersonal connections, resulting in secondary isolation (25, 26). This framework helps to illustrate how loneliness and depression mutually reinforce one another, which underscores the importance of conducting longitudinal research to capture the direction and interaction of these associations.

The literature highlights the interplay of three groups of factors—biological (vulnerability), environmental (stress), and protective—that help may help explain why some individuals are more susceptible to mental disorders and illnesses (the stress-vulnerability model). When stress levels exceed an individual’s coping resources, the likelihood of developing a mental disorder significantly increases. A supportive environment plays a protective role by helping to develop resilience. This model is also used to may help explain suicidal behavior (27). Another important—though often overlooked—aspect of men’s mental health research is the role of gender norms. Psychological literature emphasizes that traditional masculine ideals such as independence, strength, and emotional restraint can could lead to the suppression of psychological symptoms and the avoidance of psychological help (28). According to Connell’s theory of hegemonic masculinity, which describes the cultural dominance of certain forms of masculinity over others (28), and Mahalik’s Gender Role Conflict model, adopting traditional male role norms may be linked to harmful behaviors such as smoking and excessive alcohol consumption (29). Social norms that describe the health behaviors of others are a significant correlate of one’s own health behaviors. Men who experience a mismatch between societal expectations and their own emotional needs may be more vulnerable to undiagnosed mental health concerns.

The occurrence of depressive symptoms in men before the pandemic was a significant potential predictor of loneliness during its course. Depressive symptoms, especially when frequent or chronic, can strain interpersonal relationships, hinder the maintenance of social bonds, and contribute to their abandonment or social isolation (30).

In one study conducted in Canada, individuals experiencing loneliness exhibited a significant intensification of anxiety and depression symptoms. Higher levels of loneliness, anxiety, and depression were observed among individuals with lower incomes and those living alone (21). Similarly, a study conducted in Germany found that anxiety and depression were significant predictors of loneliness. It was also noted that individuals living alone demonstrated higher levels of loneliness, while those living with at least one partner experienced a reduction in loneliness levels (22). Depression symptoms present before the pandemic were the strongest potential predictor of loneliness in men during it (24).

A systematic review of Polish literature aimed at identifying factors associated with depression in adults suggests that the primary determinant of depressive disorders is the presence of a chronic illness. Among the socio-demographic factors in this group, significant determinants included age, gender, lower education level, unemployment, and low income. In the group of healthy individuals, factors associated with depression included poorer quality of life, low level and short duration of education, advanced age, and difficult material and living conditions (31). Only one of the studies included in this review, which assessed the severity of depressive symptoms, was conducted among healthy men. The prevalence of mild and moderate depression was highest in older age groups. Among the men studied, education level, when controlling for age, did not account for variance in depressive symptoms (32). It is worth noting that this data comes from a review of literature published between 2009 and 2014, and no recent findings have been identified regarding factors related to anxiety and depression in men in Poland. Longitudinal studies in the general population have shown that social bonds protect adults from depressive symptoms and disorders (33). The bidirectional relationship between depressive symptoms and loneliness may result in their simultaneous occurrence (19, 34).

Loneliness is described as an unpleasant sensation that arises when individuals perceive their social network as insufficient in both quality and quantity (35, 36). It is also defined as the discrepancy between actual and desired social relationships (37). Many definitions of loneliness characterize it as an uncomfortable state of tension, which develops over time and is closely linked to health status.

The prevalence of loneliness varies according to different studies. Data suggest that 1 in 3 adult’s experiences loneliness (38). The highest prevalence is found in Eastern Europe, with rates varying by age group: 5.9 to 9.4% among young adults, 7.7 to 12.0% in middle-aged adults, and 18.7 to 24.2% among the older adults (39). Lim et al.’s study found that 13% of participant’s experienced chronic loneliness (alludes to feelings that last longer than 2 years), while 21% experienced a loneliness episode (refers to short and infrequent feelings) (40). A study among men aged 60–64 years demonstrated that 52% felt lonely (41). During the COVID-19 pandemic, 6% of men aged 32–36 reported very high levels of loneliness, and 56% reported moderate to high levels (30).

Meta-analyses of evidence on gender differences in perceived loneliness throughout life indicate that average loneliness levels are similar for men and women (42). However, some studies, like that by Baretto et al. (37–43), found men reported higher levels of loneliness than women. In contrast, Lim et al.’s study suggested men were 11% less likely than women to report loneliness (40). The rates of loneliness among adult men are concerning, especially since over a third of the variance in suicidal thoughts and behaviors in this group appears to be associated with loneliness (44).

Loneliness appears to be associated with an approximately 30% increased risk of mortality from any may contribute to, as well as stroke and heart disease (45–49). Lonely individuals are more likely to engage in unhealthy eating behaviors, consume alcohol, and smoke tobacco (50, 51). Loneliness can also trigger symptoms of eating disorders (52). Social bond deficits in individuals aged 50–95 are linked to accelerated aging, increased morbidity, disability, and mortality (53).

According to the conceptual model, individual demographic factors—such as gender, age, ethnicity, and race—affect loneliness through structural factors like income and education, as well as through health status, stress, and social roles (related to the quality of relationships and the size of social networks) (54). Considering that loneliness, as an individual experience, occurs within a broader social context and can be shaped by it, studying socio-economic and cultural factors and their interaction with individual predictors of loneliness may be helpful in understanding cross-country differences in this research area (55). Research findings suggest that the social determinants of health (SDoH), which include non-medical factors influencing people’s health (e.g., economic systems, social policies, living conditions, social norms, income, employment, education, access to healthcare, and housing conditions), may account for 30–55% of health outcomes (56). A systematic review suggests that financial burden appears to be associated with poorer physical, mental, and functional health. Cross-sectional studies show that a higher number of depressive and anxiety symptoms is linked to greater financial burden, while longitudinal studies indicate that financial burden predicts more depressive and anxiety symptoms over time (57). It is estimated that in high-income countries, depression may contribute to the global burden of disease (GBD) to a greater extent than in low-income countries (58). Data from systematic narrative reviews of cross-sectional studies indicate that factors associated with loneliness include age (with a U-shaped distribution), female gender, socio-economic status, quality of social contacts, and the presence of chronic illnesses (23).

The socio-economic structure of Poland’s population is less favorable compared to other EU countries. The percentage of men with higher education is lower than in most EU countries. While Poland’s economic situation is improving, the GDP per capita remains one of the lowest in the EU. Economic poverty is declining, and the risk of extreme poverty decreases significantly with higher education levels. Unemployment rates in Poland are below the European average, and income disparities are narrowing (59). However, Poland is among the European countries with the lowest healthcare expenditure, at 6.4% of GDP (60).

In the context of Central and Eastern Europe, including Poland, traditional masculinity norms—such as self-reliance, dominance, and emotional restraint—remain deeply rooted in society. Research shows that men who identify with these norms are less likely to seek psychological help, which may could lead to a deterioration in their mental health and an increased risk of depression and suicide (61). Moreover, studies conducted in Poland have shown that young men often experience social pressure to conform to traditional gender roles, which may could lead to internal conflicts and difficulties in expressing emotions. Such cultural conditions may influence how men experience and express loneliness and depression, making these phenomena more difficult to identify (62).

Many countries face a deficit in care for individuals with mental health issues. Addressing this issue requires innovative approaches to diversify and enhance the level of care for these conditions. An important aspect is to engage in promotional, educational, and preventative actions (13). However, the priority should be the early identification of individuals in need of psychological or psychiatric help, and to raise public awareness that seeking such help is not associated with shame or a lower self-perception.

Our study is a screening investigation focused on identifying individuals with mental clinically relevant symptoms such as depression and anxiety, and loneliness as a social determinant of mental health, posing a risk to mental well-being. Early detection of emotional clinically relevant symptoms and the initiation of treatment are crucial from a public health perspective to prevent the exacerbation of mental health concerns in society.

The aim of the study was to assess the prevalence of anxiety symptoms, depression, irritability and the level of loneliness among men.

The following research hypotheses were examined in the study:

*H*1. Men who perceive their financial situation as worse and do not make financial decisions themselves report higher levels of depressive symptoms and loneliness compared to men in better financial situations who are in control of their finances.

*H*2. Men suffering from chronic illnesses exhibit higher levels of depressive symptoms and loneliness compared to men without such conditions.

*H*3. Men in romantic relationships experience lower levels of depressive symptoms and loneliness compared to men who are not in relationships.

*H*4. Unemployed men report higher levels of depressive symptoms and loneliness compared to employed men.

*H*5. Men with higher education experience lower levels of depressive symptoms and loneliness than men with lower levels of education.

*H*6. Men over the age of 60 exhibit higher levels of depressive symptoms and loneliness compared to younger age groups.

## 2 Materials and methods

### 2.1 Participants

The sample structure was designed to reflect demographic data based on information from the Central Statistical Office (GUS), which allows it to be considered representative within the defined strata. A total of 500 men were invited to participate in the study, of whom 438 consented and completed the questionnaire, resulting in a participation rate of 87.6%. The study was conducted using the CAWI (Computer Assisted Web Interview) internet interview technique. This is a research method in which surveys are conducted online, and respondents fill out questionnaires via the Internet, through a website dedicated to this study. The study was conducted using the random-quota sampling method. A two-stage sampling was performed, in which first localities (rural and urban) are drawn, and then a quota sample is performed. The structure of the study sample reflects the structure of the country’s population in terms of gender, age, education, size of the place of residence and province and in this approach it is representative. The sampling for the study was performed within the above-mentioned layers.

After reading the information on the purpose and course of the study, the respondent could consent to the interview. Consent was expressed by confirming it with the ‘I agree’ button on the website. Only then was it possible to start the study. Failure to confirm consent prevented the study from starting. Participation in the study was voluntary and anonymous. If the respondent changes his or her mind during the interview and does not want to continue the study, all previous answers are deleted from the system.

The study was conducted in accordance with the general principles set out in the main International ICC/ESOMAR Code, as well as with data protection requirements and other relevant legal regulations and principles set out in the national code of good research practices. Kantar complies with the principles of the PKJPA program (Polish Quality Standards for Market and Public Opinion Research). Kantar Polish annually passes an audit verifying the application of the standards included in the PKJPA program and receives the PKJPA Research Quality Certificate, among others in the CAWI category. Kantar is one of the largest companies in the world dealing with the implementation of research in many areas, including social and medical. It is also a consulting company. It has its representatives in over 100 countries around the world, including Poland.

### 2.2 Measures

#### 2.2.1 Socio-demographic questionnaire

The questionnaire collected sociodemographic data of the study group, including age, marital status, education, employment status, place of residence, income, household size, current living arrangements, as well as assessments of financial availability and financial situation. Participants were also asked about the presence of chronic diseases such as: circulatory system diseases (heart failure, coronary heart disease, hypertension, stroke, cardiac arrhythmias), respiratory system diseases (bronchial asthma, COPD, tuberculosis, cystic fibrosis), endocrine system diseases (diabetes, thyroid diseases, pancreas diseases, parathyroid diseases), neoplastic diseases, kidney diseases (chronic renal failure). The question was: Do you have any of the following chronic diseases? The respondent could indicate the answer: Yes or No.

#### 2.2.2 Hospital anxiety and depression scale (HADS-M)

To assess anxiety and depression, the study used the Polish version of the HADS–M scale (Hospital Anxiety and Depression Scale) by Majkowicz et al. (63), a modified version of the HADS (The Hospital Anxiety and Depression Scale) scale by Zigmond and Snaith (64). The HADS-M scale includes two additional statements assessing irritability levels, making a total of 16 questions, with scores ranging from 0 to 3 points per question. The maximum score for anxiety and depression (7 questions each) is 21 points, and for irritability (2 questions), it is 6 points. Scores are interpreted as follows: 0–7 indicate no disorders, 8–10 borderline, and 11–21 the presence of disorders.

In this study, the internal consistency of HADS-M was Cronbach’s alpha = 0.94 for total score, Cronbach’s alpha = 0.92 for anxiety subscale, Cronbach’s alpha = 0.88 for depression subscale and Cronbach’s alpha = 0.86 for irritability subscale.

It was a screening study using the HADS scale, which is a widely used instrument among researchers from various countries (64).

#### 2.2.3 Revised UCLA loneliniess scale (R-UCLA)

In this study, the UCLA LS loneliness scale by Russell et al. (65) in the Polish version of the Revised UCLA Loneliness Scale (R-UCLA) was used to assess the level of loneliness, after validation by Kwiatkowska et al. (66). The scale consists of 20 statements, respondents can indicate one of 4 responses (1 = I never feel this way, 4 = I often feel this way). The total scale score is the sum of the scores of the 3 subscales: belongings and affiliation, intimate others and social others (67). The maximum score that can be obtained is 80. According to the interpretation for the total scale, 4 levels of loneliness were defined: high loneliness level—65–80; moderately high loneliness level—50–64 points; moderate loneliness level—35–49 points; low loneliness level—20–34 points (68).

In this study, the internal consistency of R-UCLA was Cronbach’s alpha = 0.91 for total score, Cronbach’s alpha = 0.89 for intimate others, Cronbach’s alpha = 0.87 for social others, Cronbach’s alpha = 0.71 for belonging and affiliation.

It was a study aimed at identifying individuals at risk of loneliness, enabling early intervention to prevent the negative consequences of loneliness and to assess the effectiveness of potential therapies.

### 2.3 Procedure and ethical considerations

Research has been performed in accordance with the Declaration of Helsinki. It was voluntary for the subjects to answer the questionnaire, and they had the right to withdraw their participation at any time. All the answers were treated as strictly confidential, and the participants were guaranteed full anonymity. The subjects provided their informed consent to participate in the study. The study was approved by the Bioethics Committee at the Medical University of Warsaw (approval no AKBE/232/2020).

### 2.4 Data analysis

Normality of distributions was assessed using the Shapiro–Wilk test (for samples smaller than 100 observations), and homogeneity of variance (i.e., whether data spread is equal across groups) was tested using Levene’s test.

To compare differences between two independent groups, the parametric Student’s t-test for independent samples was used, or the Cochran–Cox test when the assumption of equal variances was not met. In cases of small and highly unequal group sizes, the nonparametric Mann–Whitney U test was applied.

To assess differences among more than two groups, a one-way analysis of variance (ANOVA) was used, or Welch’s ANOVA when the assumption of equal variances was violated, accompanied by Tukey’s *post-hoc* tests to determine between which groups the differences occurred. In cases of small and highly unequal group sizes, or when the assumptions for ANOVA were not met, the nonparametric Kruskal–Wallis test was used, along with Dunn’s post hoc test to identify the specific group differences.

To calculate effect sizes for analyses were used: *η*^2^, Eta-squared; η𝑝^2^, Partial Eta-Squared, d, Cohen’s d. Scale reliability was analyzed using Cronbach’s alpha. Calculations were performed using Statistica 10.0 software, with *p*-values ≤ 0.05 considered statistically significant.

## 3 Results

A total of 438 men took part in the study. The average age of the men in the examination was 45.61 ± 15.64 years, the youngest subject was 16 years old and the oldest was 82 years old. In the examination group, 65.8% of the subjects were in a relationship, the majority of the subjects (50.0%) had a secondary or post-secondary education, those working accounted for 64.8%. A total of 64.7% of people came from cities, 35.4% from rural areas. Most of the study respondents lived with their family (59.2%). The financial situation was rated as very good by 5.3%, rather bad by 9.4% and very bad by 2.5%. The presence of chronic diseases was indicated by 45.2% of the examination subjects. According to the HADS-M scale, a higher level of depressive symptoms was observed among men rating their financial situation as worse (*p* < 0.000), and among those with the presence of chronic illnesses (*p* < 0.001), as well as those who do not make decisions about their finances (*p* = 0.003; Table 1; Supplementary Table S1). Also, a higher score, although not statistically significant, was obtained by study subjects aged 31–40 years (M = 16.29). With regard to the level of loneliness according to the R-UCLA scale, significantly lower levels symptoms of loneliness (*p* = 0.006) were observed among respondents with secondary or post-secondary education (M = 39.72), those in a relationship (M = 38.33) compared to those who were single (M = 44.70) and those living only with a spouse/partner (M = 37.89; Table 2; Supplementary Tables S2, S4). Higher levels symptoms of loneliness were shown by those not in work and those who do not decide on their finances (M = 46.72), as well as men who rated their financial situation as very bad (M = 50.55) and rather bad (M = 42.27; Table 2; Supplementary Table S7). Detailed data is provided in Table 2 and Supplementary Tables S1–S7.

**Table 1:** Sociodemographic variables HADS-M (*n* = 438) scale scores.

Parameter	N	M	Interval –95%	Interval +95%	Med.	Min.	Max.	Q25	Q75	SD	F/H/t/Z
Age											
Up to 30 years	88	15.53	13.72	17.34	15.00	0.00	34.00	10.00	22.00	8.54	*F = 1.240* *p = 0.293* *η*_𝑝_^2^ = 0.01
31–40 years	82	16.29	14.14	18.45	17.00	0.00	39.00	7.00	25.00	9.80	
41–50 years	88	12.77	11.00	14.55	10.00	0.00	35.00	6.50	19.00	8.39	
51–60 years	83	12.43	10.24	14.63	9.00	0.00	47.00	6.00	19.00	10.06	
Over 60 years	97	12.75	10.85	14.66	10.00	0.00	42.00	6.00	21.00	9.45	
Education											
Primary or lower secondary school	25	15.92	12.33	19.51	16.00	0.00	33.00	12.00	22.00	8.71	*H = 3.389* *p = 0.335* η^2^ = 0.00
Basic education	124	14.04	12.41	15.68	12.50	0.00	40.00	7.00	21.00	9.20	
Secondary or post-secondary school	219	13.38	12.11	14.64	11.00	0.00	47.00	6.00	21.00	9.50	
Higher	70	14.67	12.42	16.92	14,00	0.00	37.00	7.00	21.00	9.44	
Marital status											
In relationship	288	13.78	12.67	14.90	11.00	0.00	47.00	6.00	21.00	9.60	*t = 0.491* *p = 0.624* d = 0.05
Single	149	14.25	12.81	15.68	13.00	0.00	35.00	7.00	21.00	8.87	
Place of residence											
Village	155	14.66	13.20	16.13	13.00	0.00	40.00	7.00	22.00	9.26	*F = 1.709* *p = 0.182* η_𝑝_^2^ = 0.01
City with up to 200,000 inhabitants	182	14.08	12.66	15.50	11.00	0.00	47.00	6.00	22.00	9.71	
City of over 200,000 inhabitants	101	12.49	10.75	14.22	10.00	0.00	42.00	6.00	17.00	8.77	
Occupational status											
Workers	267	13.75	12.66	14.84	12.00	0.00	39.00	6.00	21.00	9.03	*H = 0.060* *p = 0.996* η^2^ = 0.00
Pensioners	108	14.05	12.07	16.03	11.50	0.00	47.00	6.00	21.00	10.38	
Students	25	13.32	10.07	16.57	13.00	2.00	30.00	7.00	20.00	7.88	
Non-working	12	14.42	7.93	20.90	15.50	2.00	39.00	7.50	19.00	10.20	
With whom he/she lives											
Alone	45	13.89	11.25	16.52	14.00	0.00	29.00	7.00	21.00	8.77	*H = 0.498* *p = 0.780* η^2^ = 0.00
Only with spouse/partner	129	13.38	11.78	14.98	10.00	0.00	42.00	7.00	21.00	9.17	
With family (children/relatives)	252	14.20	13.00	15.40	12.00	0.00	47.00	6.00	21.00	9.66	
Number of persons in the household											
1 person	45	13.91	11.31	16.52	14.00	0.00	29.00	7.00	21.00	8.67	*F = 2.478* *p = 0.061* η_𝑝_^2^ = 0.02
2 persons	129	12.24	10.75	13.73	10.00	1.00	42.00	6.00	18.00	8.56	
3 persons	121	14.64	12.85	16.44	12.00	0.00	39.00	6.00	23.00	9.99	
4 persons and more	142	14.72	13.13	16.30	13.00	0.00	47.00	7.00	22.00	9.55	
Financial decision making											
Decides or co-decisions	385	13.31	12.39	14.24	11.00	0.00	47.00	6.00	20.00	9.24	*Z = 2.929* ***p = 0.003*** η^2^ = 0.02
They are not decided	46	18.33	15.57	21.08	20.50	0,00	35,00	10,00	25,00	9,29	
Incomes											
No income	13	15.46	9.43	21.50	14.00	2.00	39.00	9.00	22.00	9.99	*H = 4.739* *p = 0.315* η^2^ = 0.00
Up to 2000 zł	79	14.14	11.90	16.37	13.00	0.00	40.00	6.00	22.00	9.98	
2001–3,000 zł	97	15.27	13.33	17.20	13.00	0.00	47.00	8.00	22.00	9.60	
3,001–4,000 zł	105	13.60	11.89	15.31	12.00	1.00	37.00	6.00	21.00	8.83	
Above 4,000 zł	119	12.63	11.01	14.25	10.00	0.00	32.00	6.00	20.00	8.91	
Evaluation of the financial situation											
Very bad	11	26.09	21.02	31.16	23.00	13.00	39.00	21.00	34.00	7.54	*H = 29.137* ***p = 0.001*** η^2^ = 0.06
Rather bad	41	18.12	15.02	21.23	19.00	3.00	42.00	10.00	23.00	9.84	
Neither good nor bad	197	13.88	12.59	15.17	13.00	0.00	47.00	7.00	20.00	9.16	
Rather good	163	12.56	11.19	13.93	10.00	0.00	33.00	5.00	20.00	8.86	
Very good	23	10.22	6.83	13.61	8.00	0.00	29.00	6.00	13.00	7.84	
Presence of chronic diseases											
Has chronic diseases	198	16.35	14.92	17.79	15.50	0.00	47.00	7.00	25.00	10.24	*Z = 4.417* ***p < 0.001*** η^2^ = 0.04
Does not have	240	11.91	10.89	12.93	10.00	0.00	35.00	6.00	18.00	8.04	

N - number of participants; M, mean; Med., median; Min., minimum; Max., maximum; Q25, Q75, quartiles; SD, standard deviation; *t*, Student’s *t*-test; H, Kruskal-Wallis test; Z, Mann–Whitney U-test; F-Levene’s test; *p*, statistical significance, η^2^, Eta-squared; η𝑝^2^, Partial Eta-Squared, d, Cohen’s d.

**Table 2:** Sociodemographic variables and R-UCLA (*n* = 438) scale scores.

Parameter	N	M	Interval –95%	Interval +95%	Med.	Min.	Max.	Q25	Q75	SD	F/t//H/Z
Age											
Up to 30 years	88	44.75	42.21	47.29	46.00	24.00	69.00	35.00	52.00	12.00	*F = 2.140* *p = 0.075* η_𝑝_^2^ = 0.02
31–40 years	82	42.32	40.15	44.48	44.50	23.00	72.00	34.00	49.00	9.85	
41–50 years	88	39.88	37.59	42.16	38.50	22.00	70.00	31.00	49.00	10.78	
51–60 years	83	37.99	35.71	40.26	37.00	20.00	63.00	30.00	45.00	10.42	
Over 60 years	97	37.85	35.97	39.73	36.00	21.00	62.00	30.00	43.00	9.33	
Education											
Primary or lower secondary school	25	47.64	43.57	51.71	50.00	29.00	67.00	42.00	54.00	9.85	*H = 12.367* ***p = 0.006*** η^2^ = 0.02
Basic education	124	40.42	38.56	42.27	40.50	20.00	67.00	31.00	49.50	10.44	
Secondary or post-secondary school	219	39.72	38.32	41.12	38.00	20.00	72.00	31.00	48.00	10.50	
Higher	70	40.57	37.75	43.39	39.50	21.00	70.00	30.00	48.00	11.84	
Marital status											
In relationship	288	38.33	37.20	39.47	37.00	20.00	63.00	30.00	47.00	9.78	*t = 6.083* ***p < 0.001*** d = 0.59
Single	149	44.70	42.85	46.56	44.00	24.00	72.00	36.00	52.00	11.46	
Place of residence											
Village	155	40.81	39.03	42.58	40.00	21.00	72.00	31.00	50.00	11.18	*F = 0.923* *p = 0.391* η_𝑝_^2^ = 0.01
City with up to 200,000 inhabitants	182	40.96	39.42	42.50	41.00	20.00	69.00	32.00	49.00	10.53	
City of over 200,000 inhabitants	101	39.22	37.12	41.32	36.00	21.00	70.00	31.00	47.00	10.63	
Occupational status											
Workers	267	39.74	38.46	41.01	39.00	20.00	72.00	30.00	49.00	10.60	*H = 8.018* ***p = 0.046*** η^2^ = 0.01
Pensioners	108	39.53	37.62	41.43	38.00	20.00	62.00	32.00	48.50	10.00	
Students	25	45.56	40.54	50.58	46.00	24.00	67.00	36.00	50.00	12.17	
Non-working	12	46.67	37.58	55.75	47.50	29.00	66.00	30.50	57.50	14.30	
With whom he/she lives											
Alone	45	43.29	39.69	46.89	41.00	21.00	72.00	33.00	51.00	11.98	*H = 9.236* ***p = 0.010*** η^2^ = 0.02
Only with spouse/partner	129	37.89	36.39	39.39	37.00	20.00	63.00	31.00	44.00	8.61	
With family (children/relatives)	252	41.10	39.70	42.51	41.00	20.00	70.00	31.00	50.00	11.34	
Number of persons in the household											
1 person	45	43.87	40.36	47.37	42.00	21.00	72.00	37.00	51.00	11.66	*F* = 5.092 ***p = 0.002*** η_𝑝_^2^ = 0.03
2 persons	129	38.13	36.56	39.70	36.00	23.00	67.00	31.00	44.00	9.00	
3 persons	121	41.62	39.53	43.71	42.00	20.00	70.00	31.00	50.00	11.62	
4 persons and more	142	40.70	38.88	42.51	40.00	22.00	67.00	31.00	50.00	10.93	
Financial decision making											
Decides or co-decisions	385	39.71	38.65	40.78	38.00	20.00	72.00	31.00	48.00	10.59	*Z = 3.435* ***p < 0.001*** η^2^ = 0.04
They are not decided	46	46.72	43.50	49.93	49.00	29.00	70.00	37.00	52.00	10.83	
Incomes											
No income	13	47.38	38.79	55.98	50.00	29.00	67.00	31.00	57.00	14.22	*H = 16.267* ***p = 0.002*** η^2^ = 0.03
Up to 2000 zł	79	42.44	39.86	45.02	41.00	24.00	69.00	32.00	50.00	11.52	
2001–3,000 zł	97	42.49	40.34	44.65	42.00	23.00	72.00	34.00	50.00	10.71	
3,001–4,000 zł	105	38.15	36.11	40.20	36.00	20.00	67.00	30.00	47.00	10.58	
Above 4,000 zł	119	38.53	36.78	40.28	38.00	20.00	58.00	30.00	47.00	9.63	
Evaluation of the financial situation											
Very bad	11	50.55	44.76	56.33	53.00	38.00	66.00	41.00	57.00	8.61	*H = 13.575* ***p = 0.009*** η^2^ = 0.03
Rather bad	41	42.27	38.91	45.63	42.00	26.00	63.00	32.00	50.00	10.65	
Neither good nor bad	197	41.08	39.51	42.65	41.00	21.00	72.00	32.00	49.00	11.15	
Rather good	163	38.93	37.36	40.49	38.00	20.00	67.00	31.00	47.00	10.11	
Very good	23	39.30	34.61	44.00	41.00	23.00	57.00	30.00	50.00	10.85	
Presence of chronic diseases											
Has chronic diseases	198	41.23	39.76	42.71	40.50	20.00	70.00	32.00	50.00	10.52	*Z = 1.617* *p = 0.106* η^2^ = 0.04
Does not have	240	39.90	38.51	41.30	39.00	20.00	72.00	31.00	48.00	10.99	

N - Number of participants; M, mean; Med., median; Min., minimum; Max., maximum; Q25, Q75, quartiles; SD, standard deviation; F-Levene’s test; *t*, Student’s *t*-test; H, Kruskal-Wallis test; Z, Mann–Whitney U-test; *p*, statistical significance, η^2^, Eta-squared; η𝑝^2^, Partial Eta-Squared, d, Cohen’s d.

On the HADS-M scale, participants scored an average of 13.91 ± 9.35. The average scores on the anxiety subscale were 6.51 ± 4.29, on the depression subscale 5.14 ± 4.29, and on the irritability subscale, the average scores were 7.39 ± 5.26. On the loneliness scale, the average score was 40.50 ± 10.78 (Table 3).

**Table 3:** Presents data on anxiety, depression, irritability measured by the HADS-M scale, and loneliness assessed by the R-UCLA scale.

Parameter	M	Interval –95%	Interval +95%	Med.	Min.	Max.	Q25	Q75	SD
Anxiety subscale	6.51	6.09	6.95	5.00	0.00	21.00	3.00	10.00	4.55
Depression subscale	5.14	4.74	5.54	4.00	0.00	20.00	1.00	8.00	4.29
Irritability subscale	2.25	2.10	2.42	2.00	0.00	6.00	1.00	4.00	1.71
Total score	13.91	13.04	14.80	12.00	0.00	47.00	6.00	21.00	9.35
R-UCLA									
Belonging and affiliation	9.58	9.30	9.87	9.00	5.00	20.00	7.00	12.00	3.03
Intimate others	22.32	21.71	22.95	21.00	10.00	40.00	17.00	26.00	6.61
Social others	8.59	8.28	8.90	8.00	5.00	20.00	6.00	11.00	3.30
Total score	40.50	39.49	41.52	40.00	20.00	72.00	31.00	49.00	10.78

M, mean; Med., median; Min., minimum; Max., maximum; Q25, Q75, quartiles; SD, standard deviation.

In the HADS-M scale, 20.77% of men were found to have depressive symptoms, and 16.21% were in the borderline state. Anxiety depressive symptoms were identified in 21.91% of participants, with 13.92% borderline. In the depression subscale, 12.55% had depressive symptoms and 18.49% were borderline. Irritability depressive symptoms were noted in 27.62%, with 13.47% borderline. Low levels symptoms of loneliness were observed in 35.16% of participants, moderate in 41.55%, moderately high in 21.00%, and very high in 2.30% (Table 4).

**Table 4:** Participants’ may result in the HADS-M scale; R-UCLA.

Scale	Parameter		n	%
HADS-M	Anxiety Subscale	No Symptoms Disorders	281	64.15
		Borderline states	61	13.92
		Presence Symptoms of Disorders	96	21.91
	Depression Subscale	No Symptoms Disorders	302	68.94
		Borderline states	81	18.49
		Presence Symptoms of Disorders	55	12.55
	Irritability Subscale	No Symptoms Disorders	272	62.10
		Borderline states	45	10.27
		Presence Symptoms of Disorders	121	27.62
	Total score	No Symptoms Disorders	276	63.01
		Border conditions	71	16.21
		Occurrence Symptoms of Disorders	91	20.77
R-UCLA	Total score	Low level of loneliness	154	35.16
		Moderate level of loneliness	182	41.55
		Moderately high level of loneliness	92	21.00
		High level of loneliness	10	2.30

Four significant predictors of anxiety, depression, and irritability (HADS-M) were identified: age, self-assessed financial situation, presence of chronic illness, and the total loneliness score (R-UCLA). The multiple coefficient of determination (R^2^) indicated that approximately 35% of the variance in the dependent variable HADS-M Total was may help explain by the model. The overall model was statistically significant. The combined correlation of all variables (multiple R) with the HADS-M Total score was 0.580, indicating a moderate correlation (Table 5).

**Table 5:** Test of SS whole model vs. SS residual.

Dependent variable	Multiple R	Multiple R^2^	Adjusted R^2^	SS model	df model	MS model	SS residual	df residual	MS residual	F	*p*-value
HADS-M total	0.580	0.337	0.315	11121.602	12	926.800	21887.828	366	59.803	15.498	**<0.001**

Dependent Variable—the outcome variable; Multiple R, Multiple Correlation Coefficient; Multiple R^2^, Multiple Coefficient of Determination, Adjusted R^2^, Adjusted Coefficient of Determination; SS Model, Sum of Squares for the Model; df Model, Degrees of Freedom for the Model; MS Model, Mean Square for the Model; SS Residual, Sum of Squares for Residuals; df Residual, Degrees of Freedom for Residuals; MS Residual, Mean Square for Residuals; F, statistic value; *p*, level of statistical significance.

**Figure 1:**
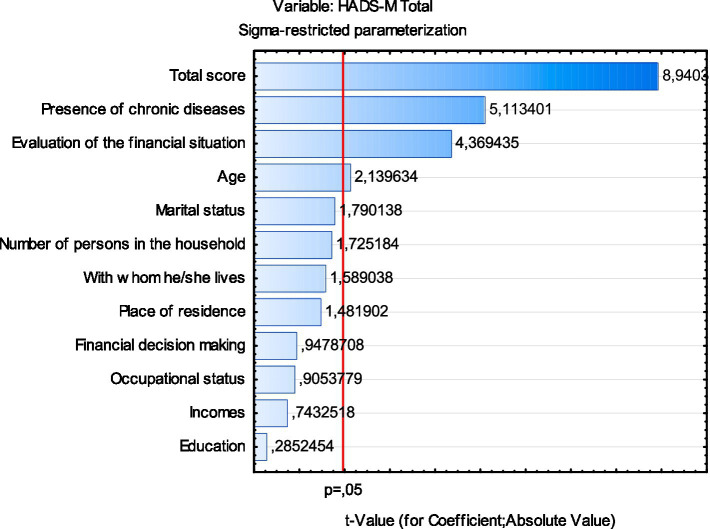
Chart of *t*-values for coefficients; df = 366.

As shown in Figure 1, the strongest statistically significant predictors of the HADS-M Total score were: the total R-UCLA score, presence of chronic illness, financial situation assessment, and age.

Four significant predictors of loneliness (R-UCLA) were identified: age, employment status, marital status, and the total HADS-M score (Table 6). The multiple coefficient of determination (R^2^) indicated that approximately 34% of the variance in the dependent variable R-UCLA was may help explain by the model. The overall model was statistically significant. The combined correlation of all variables (multiple R) with the loneliness variable was 0.579, indicating a moderate correlation.

**Table 6:** Test of SS whole model vs. SS residual.

Dependent variable	Multiple R	Multiple R^2^	Adjusted R^2^	SS model	df model	MS model	SS residual	df residual	MS residual	F	*p*-value
R-UCLA total	0,579	0.335	0.313	15074.941	12	1256.245	29965.566	366	81.873	15.344	**<0.001**

Dependent Variable—the outcome variable; Multiple R, Multiple Correlation Coefficient; Multiple R^2^, Coefficient of Determination, R^2^; Adjusted R^2^, Adjusted Coefficient of Determination; SS Model, Sum of Squares for the Model; df Model, Degrees of Freedom for the Model; MS Model, Mean Square for the Model; SS Residual, Sum of Squares for Residuals; df Residual, Degrees of Freedom for Residuals; MS Residual, Mean Square for Residuals; F, statistic value; *p*, level of statistical significance.

**Figure 2:**
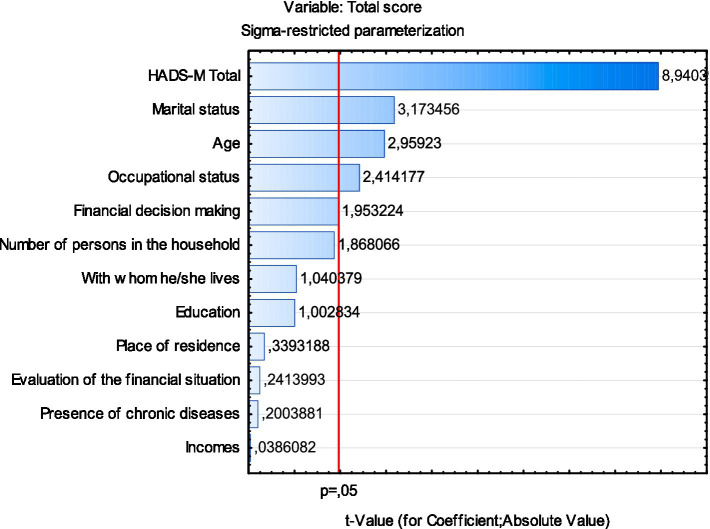
Chart of *t*-values for coefficients; df = 366.

As shown in Figure 2, the strongest statistically significant predictors of the total loneliness score (R-UCLA) were: the HADS-M total score, marital status, age, and employment status.

## 4 Discussion

This study provides new evidence in the field of men’s mental health. The results of this study may help to build strategies to support men’s mental health. The aim of this study was to assess the prevalence of anxiety symptoms, depression, irritability and to assess the level of loneliness among men in Poland. In this study depression symptoms were observed in 12.5% of participants, with 18.49% in borderline states. Anxiety was present in 21.91% of men, with 13.92% in borderline states. Irritability affected 27.62% of men, with 10.27% in borderline states.

Similar depression rates were found in other studies, including one in Germany where 12.2% of men demonstrated major depression symptoms. The anxiety level in this study was lower compared to our observations. Mild anxiety symptoms affected 23.8% of men, moderate anxiety 6.2%, and severe anxiety 4.4% (69). According to the results of studies by other authors conducted in Poland, with the outbreak of the pandemic and during its duration, the average level of depression symptoms in men increased significantly, with the highest average in 2022 (M = 13.16). In the group of women and men, the percentage of people experiencing mild depression also decreased, and the percentage of people with moderate (47.6%), moderately severe (20.5%) and severe depression (6.4%) increased significantly in 2022, classified according to the recommended depression screening tool - Patient Health Questionnaire PHQ-9 (11). In the study by Zalewska et al. with the participation of 40.2% of men, in total symptoms of moderate or severe depression appeared in 23 and 2.7% of participants (70). In the study by Zwolińska et al., 44% of respondents demonstrated depressive symptoms, including 1.3% symptoms of severe depression according to the Beck Depression Inventory (71).

Reports from other European countries are varied. In Italy, for the general population, moderate, high, and very high levels of depression symptoms were observed in 67.2%; 17%; 15.8% of the study participants, respectively (72), while in one study in Spain, a total of 18.7% demonstrated depressive symptoms (73). In another study, this percentage for the male population ranged from mild to severe to moderate, respectively, 8.7%; 4.0%; 2.9% (74).

For comparison in a group of Chinese men, moderate to severe depression was noted in 12.5% of participants, with mild depression symptoms in 17.6% of male individuals. Meanwhile, mild anxiety symptoms were experienced by 20.9% of men, with moderate to severe anxiety in 11% (75). In research that included Iranian men, 16.1% suffered from severe anxiety, and 20.3% indicated a moderate level of anxiety (76). Pradeepa’s study observed that anxiety in men was positively associated with an increased intensity of depression symptoms (77).

In our literature search, we found one of the few studies showing that the risk of mental disorders such as anxiety and depression was more prevalent in men than in women, significantly associated with financial issues and income levels (78). Arias - de la Torre and colleagues in their population study across 27 European countries (including Poland) highlighted the impact of income on depression symptoms, noting a decrease in depression with higher income (79). Also according to data presented by the Public Opinion Research Center in Poland, the most negative opinions about one’s mental health were recorded in groups of respondents who described their financial situation as bad, among the unemployed, pensioners and pupils and students. Depression also co-occurs noticeably with low economic status. The data show that depression was experienced slightly more often than average by people with low income per person in the household (from PLN 1.000 to PLN 1.500–15%) and those who assessed their financial situation as bad (14%) (80).

Comparisons with other European countries indicate that, although depressive symptoms are statistically more common among women, the magnitude of this difference is not uniform across Europe. Van de Velde et al. demonstrated that this gender gap is significantly wider in Eastern and Southern European countries, suggesting that cultural differences—particularly those related to gender norms and social expectations regarding male and female roles—play an important role in shaping how individuals experience and express their mental state (81). In countries where traditional masculinity norms dominate, men may be less likely to recognize and report depressive symptoms, which contributes to delayed diagnosis. In countries such as Poland, strong social expectations persist regarding men’s roles as self-reliant, emotionally restrained, and dominant family leaders. The pressure placed on men in this region to fulfill these norms is often associated with reluctance to seek psychological help and suppression of emotional distress, which can worsen their mental health (62). Due to cross-national differences, not only do depression rates vary, but so do the ways in which men experience and express mental health concerns, depending on the cultural context. In countries where men are encouraged to express emotions—such as in Scandinavian countries—rates of depression among men are significantly lower. In Central and Eastern Europe, where masculinity norms are more conservative, men are less likely to seek mental health support and often ignore or downplay their psychological struggles, which can could lead to deeper isolation and deteriorating mental health (61). Cultural differences in the underlying may be associated with of irritability may also contribute to its varying prevalence. Greater irritability may be observed in communities with fewer financial resources, due to challenges in meeting basic physiological needs. Culturally influenced habits related to sleep or nutrition may also contribute to differences in the prevalence of irritability (82).

Another significant factor exacerbating depression symptoms in men was chronic illness (66–74). In our study, higher levels of depression and anxiety were found in men who rated their financial situation as very poor, those who had no control over their finances, and individuals dealing with any chronic disease. Financial issues or physical illnesses in men can challenge traditional masculine ideals, leading to feelings of failure since societal expectations often dictate that men should be strong and successful. Despite changing social views, often from a young age, boys are taught that expressing sadness or crying is shameful, leading to a tendency among men to avoid seeking medical help for emotional issues related to depression, rarely discussing their feelings with close ones, and often not acknowledging their problems, complicating diagnosis when they do consult a doctor (83). In recent years, there have been more calls for the normalization of men’s emotionally, which can help improve their mental health. However, there still a need to break these norms through education, so that crying in men is not seen as a sign of weakness, but as a healthy form of expressing emotions.

In our project, we not obtained statistically significant results regarding age, education, marital status, or the participants’ living areas. However, we found that the worst depression symptoms appeared in men aged 31 to 40. This result was surprising to researches, because many authors indicate an increase in the percentage of depression symptoms with age (79). However, we would like to cite the results of one of the Polish studies conducted among young men in Poland, where over 40% of participants had depressive symptoms above the norm, and 49.36% had anxiety symptoms above the norm. The average age of participants in that study was 24.8 ± 3.75 (84).

It was also noted that men with lower education, who were single, and living in rural areas, faced these issues more frequently. In a study by Zhou et al., low education, rural living, and poor economic status in a group of Chinese men correlated with higher depression symptoms, with slightly higher rates in men (17.6%) compared to women (17%) (85). Similar findings regarding education levels were reported by other authors, with depression symptoms intensifying with age (79, 86). A study focusing on rural Chinese men observed higher depression rates among unmarried individuals, worsening with age, especially in the younger demographic of 20 to 40 years, which aligns with the age group in our study showing the highest depression symptom levels (87). Zhou et al.’s subsequent research confirmed the highest depression rates among single men with lower education and financial status (88).

Research on loneliness and depression shows a two-way relationship between them, which can be may help explain by the vicious cycle of loneliness model. As outlined in the introduction to this study, the model assumes that loneliness activates negative cognitive schemas, leading individuals to withdraw from social interactions, which in turn intensifies isolation and depressive symptoms. Loneliness is a unique psychological state in which an individual perceives themselves as socially disconnected—even when surrounded by others. In this way, loneliness and depression reinforce one another, resulting in the gradual worsening of symptoms. This dynamic may help may help explain the co-occurrence of these phenomena observed in our findings (25).

Higher levels of loneliness predict the occurrence of depressive symptoms in the future and vice versa, where loneliness can adversely affect the course of depression (24, 89). Studies during the COVID-19 pandemic confirmed that loneliness is a significant factor in depressive and anxious symptoms, particularly among men and younger people. The relationship between loneliness and depression symptoms was stronger in men compared to women (90). Gender was also negatively related to loneliness, with men reporting it more frequently than women (43, 91), although a study by Lee et al. found a significantly lower level of loneliness in men (36%) compared to women (64%) (24). From a socio-cultural perspective, it is important to refer to the concept of hegemonic masculinity. According to this theory (Connell et al.), dominant masculinity norms promote self-reliance, emotional restraint, and avoidance of admitting vulnerability. Such patterns may limit men’s ability to seek emotional support and contribute to the development of loneliness and symptoms of undiagnosed depression, particularly in the context of difficult socio-economic conditions (28). In reference to the vulnerability–stress model, the socio-environmental factors identified in this study help to may help explain their role in activating existing predispositions to mood disorders (92). According to the self-determination theory, the inability to meet basic psychological needs—autonomy, competence, and relatedness—can could lead to a decline in mental well-being (93).

This study did not find a significant relationship between participants’ age and their perception of loneliness. The highest levels of loneliness were observed in men up to the age of 30, a finding also supported by Hawkley et al.’s (94) research on age differences in loneliness among genders in the United States. In the German population, 20.8% of men aged 16–24 and 17.1% of those aged 25–34 reported feeling lonely, with the highest loneliness rates in the 45–54 age group (95).

The narrative review suggests that marital status, living conditions, and characteristics of personal social networks are considered predictors of loneliness both before and during the COVID-19 pandemic (96). In this study, loneliness perception was higher among single men. Reinwarth et al.’s research demonstrated significantly lower loneliness among men in relationships (13%) compared to those not in relationships (30%). The highest levels of loneliness were reported by unmarried men aged 55–64 (95). Living without a spouse or partner among individuals aged 50–70 was more strongly associated with loneliness in men than in women. (97). Research suggests that being in a relationship offers greater protective benefits against loneliness for men than for women (96). In men, literature has confirmed a negative correlation between perceived loneliness and household size (96, 98). In this study, the least lonely men were those living in two-person households and those living only with a spouse/partner. Conversely, the study found that living with children or other family members does not protect against loneliness, a finding also noted by other researchers (99, 100).

Living in rural areas is considered a risk factor for loneliness (91, 101). The study did not confirm significant differences in the perception of loneliness based on men’s place of residence. In this study, men with basic education, no income, and those who rated their financial situation as very poor perceived loneliness the highest. In the overall Dutch sample, it was shown that risk factors associated with higher levels of loneliness were being male, lower level of education, and inadequate financial resources (102). The demographic variables that were significant for loneliness in the general population in Slovenia were marital status and employment status (91). Completing higher education was observed to be associated with approximately an 8% difference in the likelihood of loneliness among men in the study by Kung et al. (98). A study conducted in the United States also demonstrated that loneliness is best may help explain, among other factors, by household income (103). Data collected from 14 European countries confirmed that among men aged over 65, the prevalence of loneliness was higher among the least affluent (22.08%) and decreased with increasing affluence (104). A review of studies suggests that a deterioration in financial situation likely may could lead to a greater sense of loneliness, particularly among middle-aged and older adults. This relationship appears to be dependent on age and country of residence. Higher levels of loneliness among older adults, including in Poland, may be linked to socio-economic conditions and poor health. Research suggests that the relationship between financial situation and loneliness is moderated by health and the availability of social networks (96).

This study highlighted the highest levels of loneliness among unemployed men. Research in higher-income countries shows a correlation between loneliness and unemployment, with the association growing stronger with increased loneliness, peaking at ages 30–34 and 50–59. Unemployment has been linked to at least a 40% increase in the likelihood of reporting loneliness (105). Gender differences did not significantly impact the relationship between loneliness and employment status (106). In the United Kingdom, unemployed men are estimated to have a 3.1% higher chance of feeling lonely compared to those who are employed (98). Autonomy and environmental mastery are dimensions of well-being. Data suggests that the strongest potential predictor of loneliness among men was environmental mastery (30). Men with a elevated level of environmental mastery may be more inclined to seek social support, which can reduce the risk of loneliness.

Given that loneliness is a significant public health issue, it is crucial to identify which subgroups of men are more at risk due to sociodemographic factors. In daily medical practice, it is essential to consider risk factors that contribute to the increase in mental health concerns among men. According to our findings, these include economic factors. It is recommended that the assessment of socio-economic conditions and screening for depression and loneliness, particularly among individuals with chronic illnesses, become an integral part of the diagnostic and care process carried out by primary care physicians, nursing staff, and mental health specialists. Early identification of financial problems can facilitate referring patients to appropriate support within the social care system, such as financial counseling or assistance programs.

In Poland, men constitute only 27% of those utilizing National Health Fund services for the diagnosis and treatment of depression (10). It is advisable to design and target mental health campaigns specifically for men. Preventive measures should include providing men with knowledge, developing skills, and building competencies for identifying mental health issues and managing them effectively. To reach a larger number of men, these initiatives should be implemented in places frequently visited by men, such as workplaces, sports centers, public transportation, bars, and restaurants (107). Strategies for preventing depression among men should also promote reducing loneliness and ensuring access to quality mental health care when needed.

At the systemic level, it is essential to implement policies that mitigate the impact of financial uncertainty on mental health. The National Mental Health Protection Program for 2023–2030 emphasizes the need to integrate healthcare with social assistance and to promote mental health initiatives in the workplace. Introducing financial support programs for low-income individuals and providing financial education can help reduce stress related to economic challenges (108).

An example of interventions tailored to male roles and gender-related expectations are the “Movember” and “Heads Up” campaigns, which engage athletes and celebrities in promoting mental health awareness (109, 110). The Movember campaign is an international initiative that encourages men to grow mustaches during the month of November to raise awareness about men’s health, including mental health. The campaign involves athletes and public figures in promoting open discussions about depression, anxiety, and other mental health issues, aiming to break the stigma surrounding help-seeking among men. In Poland as well, November is a month of solidarity with men battling prostate cancer, testicular cancer, and depression. The Heads Up campaign is a British initiative in collaboration with the Premier League, aimed at raising awareness of mental health, particularly among men. The campaign features well-known football players who share their personal experiences with depression and stress, encouraging open conversations about emotional difficulties and the importance of seeking support.

### 4.1 Limitations

Our study, however, had certain limitations. Due to its cross-sectional design, the study provides a snapshot of men’s mental health at a specific point in time and limits the ability to draw causal conclusions. Although associations were observed between variables such as financial situation, chronic illness, relationship status, and mental health symptoms, a cross-sectional study does not allow us to determine whether these factors directly may contribute to or merely co-occur with mental health concerns. Additionally, it is difficult to establish the direction of the observed relationships—for example, whether depression may could lead to a worse financial situation or vice versa. Furthermore, the findings may not be fully generalizable to other populations or settings, particularly those that differ culturally, socially, or demographically. The surveys were filled out and sent back through the internet, potentially excluding potential respondents without internet technology access or with limited knowledge on how to use it. Moreover, differences between our study’s results and those of other studies could be due to methodological differences related to the use of different scales. It should also be noted that the HADS-M and R-UCLA scales assess only some aspects of mental well-being and are used in screening studies. The study has its strengths, including the use of random sampling. The sample structure mirrors the demographic composition of the country’s population in terms of gender, age, education, size of the place of residence, and region, making it representative of the studied population. There is also evidence suggesting that online surveys tend to have higher disclosure rates for sensitive topics (111) and a higher level of data reliability, possibly due to reduced privacy concerns (112). Future research directions should emphasize the need for longitudinal studies to investigate the temporal relationships between loneliness, anxiety, depression, and socio-economic factors in this population.

## 5 Conclusion

In this study, one fifth of participants demonstrated anxiety symptoms disorders, and every tenth man had symptoms of depressive anxiety symptoms disorders. These were more prevalent among men with a negative view of their financial situation, those not in control of their finances, individuals with chronic diseases, and younger men.

Every fifth man experienced a moderately elevated level of loneliness. Higher level of loneliness was more prevalent among men with lower education, who were single, living alone, unemployed, with a negative assessment of their financial situation, and lacking financial decisiveness.

Screening studies for mental well-being assessment among men should continue and be a fundamental part of public health due to the lower detectability of mental health issues and the higher rate of successful suicides in the male group.

The results of this study confirm the need for screening tests aimed at men on a broader scale than before, as well as social campaigns aimed at shaping social attitudes toward mental health concerns and the feeling of loneliness in men. Future studies should be designed as longitudinal studies, thanks to which it will be possible to obtain information about the mental health status of men over a longer period of time and information about whether they are chronic or situational.

## Data Availability

The original contributions presented in the study are included in the article/Supplementary material, further inquiries can be directed to the corresponding author.
