# Supplementary material for: Loneliness and depression among men in Poland: cross-sectional study
Source: Front Public Health. 2025 Jun 17;13:1539822. doi: 10.3389/fpubh.2025.1539822 (PMC12209323; doi:10.3389/fpubh.2025.1539822)
Supplement: Supplementary file 1 [file Data_Sheet_1.pdf]

Supplementary Material

Table S1 Results of the HADS-M post-hoc test between groups in relation to the assessment of the financial situation

**HADS-M**	Kruskal-Wallis test: H =29.137, p = .0000				
Evaluation of the financial situation	Very bad	Rather bad	Neither good nor bad	Rather good	Very good
	R:365.23	R:271.84	R:218.50	R:200.76	R:169.52
Very bad		0.287	**0.002**	**0.000**	**0.000**
Rather bad	0.287		0.134	**0.012**	0.018
Neither good nor bad	**0.002**	0.134		1.000	0.771
Rather good	0.000	0.012	1.000		1.000
Very good	0.000	0.018	0.771	1.000	

Table S2 Post-hoc R-UCLA test results between groups with respect to education

**R-UCLA**	Kruskal-Wallis test: H =12.366, p =.0062			
Education	Primary or lower secondary school	Basic education	Secondary or post-secondary school	Higher
	R:304.14	R:220.09	R:210.36	R:216.82
Primary or lower secondary school		**0.015**	**0.003**	**0.018**
Basic education	**0.015**		1.000	1.000
Secondary or post-secondary school	**0.003**	1.000		1.000
Higher	**0.018**	1.000	1.000	

Table S3 Results of the R-UCLA post-hoc test between groups in relation to professional situation

**R-UCLA**	Kruskal-Wallis test: H =8.018, p =.0456			
Occupational status	Workers	Pensioners	Students	Non-working
	R:201.34	R:201.16	R:258.06	R:261.96
Workers		1.000	0.136	0.507
Pensioners	1.000		0.187	0.560
Students	0.136	0.187		1.000
Non-working	0.507	0.560	1.000	

Table S4 Post-hoc test results of R-UCLA between groups with respect to living with other people

**R-UCLA**	Kruskal-Wallis test: H =9.236, p =.0099		
With whom he/she lives	Alone	Only with spouse/partner	With family (children/relatives)
	R:244.21	R:188.11	R:221.01
Alone		**0.025**	0.733
Only with spouse/partner	**0.025**		**0.041**
With family (children/relatives)	0.733	**0.041**	

Table S5 Results of the R-UCLA post-hoc test between groups with respect to the number of persons in the household

**R-UCLA**	HDS test			
Number of persons in the household	1 person	2 persons	3 persons	4 persnons and more
	M=43.867	M=38.132	M=41.620	M=40.697
1 person		0.053	0.751	0.495
2 persons	0.053		0.054	0.216
3 persons	0.751	0.054		0.908
4 persons and more	0.495	0.216	0.908	

Table S6 Post-hoc test results of R-UCLA between groups with respect to income

**R-UCLA**	Kruskal-Wallis test: H =16.267, p = .0027				
Incomes	No income	Up to 2000 zł	2001-3000 zł	3001-4000 zł	Above 4000 zł
	R:269.00	R:226.06	R:230.72	R:182.29	R:190.05
No income		1.000	1.000	0.135	0.236
Up to 2000 zł	1.000		1.000	0.138	0.376
2001-3000 zł	1.000	1.000		**0.040**	0.127
3001-4000 zł	0.135	0.138	**0.040**		1.000
Above 4000 zł	0.236	0.376	0.127	1.000	

Table S7 Results of the R-UCLA post-hoc test between groups in relation to self-assessed financial situation

**R-UCLA**	Kruskal-Wallis test: H =13.575, p = .0088				
Evaluation of the financial situation	Very bad	Rather bad	Neither good nor bad	Rather good	Very good
	R:333.36	R:239.15	R:221.97	R:201.57	R:207.59
Very bad		0.273	**0.042**	**0.008**	0.064
Rather bad	0.273		1.000	0.871	1.000
Neither good nor bad	**0.042**	1.000		1.000	1.000
Rather good	**0.008**	0.871	1.000		1.000
Very good	0.064	1.000	1.000	1.000	
